# Extracellular vesicles of trypomastigotes of *Trypanosoma cruzi* induce changes in ubiquitin-related processes, cell-signaling pathways and apoptosis

**DOI:** 10.1038/s41598-023-34820-6

**Published:** 2023-05-10

**Authors:** Alberto Cornet-Gomez, Lissette Retana Moreira, Thales Kronenberger, Antonio Osuna

**Affiliations:** 1 Grupo de Bioquímica y Parasitología Molecular (CTS 183), Departamento de Parasitología, Instituto de Biotecnología, Universidad de Granada, Campus de Fuentenueva, 18071 Granada, Spain; 2 Departamento de Parasitología, Facultad de Microbiología, Universidad de Costa Rica, San José, 11501 Costa Rica; 3 Centro de Investigación en Enfermedades Tropicales (CIET), Universidad de Costa Rica, San José, 11501 Costa Rica; 4 Institute of Pharmacy, Pharmaceutical/Medicinal Chemistry and Tübingen Center for Academic Drug Discovery (TüCAD2), Eberhard Karls University Tübingen, Auf der Morgenstelle 8, 72076 Tübingen, Germany; 5 School of Pharmacy, Faculty of Health Sciences, University of Eastern Finland, 70211 Kuopio, Finland

**Keywords:** Biochemistry, Cell biology, Microbiology, Diseases, Molecular medicine

## Abstract

Chagas disease is caused by the protozoan parasite *Trypanosoma cruzi*. The disease has an acute and a chronic phase in which approximately 30% of the chronic patients suffer from heart disease and/or gastrointestinal symptoms. The pathogenesis of the disease is multifactorial and involves the virulence of the strains, immunological factors and extracellular vesicles (EV) shed by the parasite which participate in cell–cell communication and evasion of the immune response. In this work, we present a transcriptomic analysis of cells stimulated with EV of the trypomastigote stage of *T. cruzi*. Results after EV-cell incubation revealed 322 differentially expressed genes (168 were upregulated and 154 were downregulated). In this regard, the overexpression of genes related to ubiquitin-related processes (*Ube2C*, *SUMO1* and *SUMO2*) is highlighted. Moreover, the expression of Rho-GTPases (*RhoA*, *Rac1* and *Cdc42*) after the interaction was analyzed, revealing a downregulation of the analyzed genes after 4 h of interaction. Finally, a protective role of EV over apoptosis is suggested, as relative values of cells in early and late apoptosis were significantly lower in EV-treated cells, which also showed increased CSNK1G1 expression. These results contribute to a better understanding of the EV-cell interaction and support the role of EV as virulence factors.

## Introduction

*Trypanosoma cruzi* is an intracellular protozoan parasite, the etiologic agent of American trypanosomiasis or Chagas disease. The World Health Organization (WHO) estimates 6–7 million people to be infected and approximately 75 million people are at risk of infection with this parasite worldwide. Chagas disease is endemic in Latin America due to the presence of blood-sucking triatomine bugs (Hemiptera, Reduviidae) that transmit the parasite to vertebrate hosts^1^. However, infection can also occur through blood transfusions and organ transplantation, through vertical transmission and after consumption of food/drinks contaminated with the excreta/feces of infected triatomines. Nowadays, Chagas disease has a global expansion due to human displacements, emerging in areas of the United States, Canada, Europe, Australia and Japan, considered disease-free a few decades ago^2^. Despite its prevalence and severity, there is neither a truly specific treatment for the chronic phase nor effective prophylaxis for this disease^3^.

*T. cruzi* presents a complex life cycle that includes hematophagous reduviid bugs (invertebrate hosts) and mammalian vertebrate hosts. The life cycle starts when the insect takes a blood meal from a vertebrate host that contains the blood trypomastigote stage of the parasite. These forms migrate to the midgut of the insect and differentiate into epimastigotes, a highly replicative stage inside the invertebrate host. After 8–15 days after blood ingestion, some of these epimastigotes will transform in the final part of the insect´s intestine and rectal ampulla into non-proliferative, metacyclic trypomastigotes. Parasites will be released with its feces when feeding on a vertebrate host. Metacyclic trypomastigotes could gain access to the vertebrate host by skin discontinuities and invade a wide variety of nucleated cells. Inside the cells, these forms differentiate into amastigotes, the replicative stage of the parasite inside the vertebrate host, and after 96–120 h (and several rounds of multiplication), amastigotes will differentiate into trypomastigotes and lyse the cells. After reaching the bloodstream^4^, trypomastigotes will be able to infect other cells and are also available for their ingestion by other triatomines, thus completing their life cycle^5^.

Chagas disease displays symptomatic and pathological variations among the infected individuals. Moreover, the pathogenesis of the disease is considered a multifactorial process that involves many interactive pathways^6^. In this sense, the molecular invasion mechanisms by trypomastigotes of *T. cruzi* and the associated regulatory pathways have been intensely investigated for many years^7^. A large number of secreted molecules have been suggested to be involved in host cell invasion mechanisms, and some of them have been described to be associated with extracellular vesicles^8,9^.

Extracellular vesicles (EV) are small membrane-bound vesicles released to the extracellular milieu by almost any type of cell and can be classified based on their size, biogenesis and composition; this classification includes: (a) exosomes (20–100 nm), (b) ectosomes (100–1000 nm) and (c) apoptotic blebs (> 1000 nm), among others^8,9^. EV composition is complex and proteins, lipids, nucleic acids (DNA and RNA) and/or active metabolites are part of the EV cargo^8–11^. The secretion of EV by *T. cruzi* was first demonstrated by da Silveira et al. in 1979^12^; since then, several research groups have been studying the role of EV in the pathogenesis of Chagas disease, demonstrating significant effects mainly in cell–cell communications, cell infection and evasion of the immune response^9,11,14–16^.

EVs secreted by different stages of *T. cruzi* contain several surface components that are involved in the adhesion, invasion and even migration of epimastigote forms as the parasite migrates along the vector’s gut^13–17^. Previous studies performed by our research group have also demonstrated the role of EV of trypomastigotes in generating changes in cell physiology, such as cell membrane permeability, increases in intracellular free calcium concentrations, disruption of the cytoskeleton or cell cycle functionality which facilitate cell parasitization^18,19^. Nowadays, there are many descriptions of the effects of EV on *T. cruzi* over host cells, and big data analyses such as RNA analysis, proteomics and transcriptomics can help explain these changes at molecular scale.

In order to delve into the effects of EV over cells at transcriptome level, we present a transcriptomic analysis of Vero cells stimulated with EV secreted by the trypomastigote stage of *T. cruzi* (the infective stage of the parasite to the vertebrate host) and reveal the differential expression of genes after the stimulation. The results obtained contribute to a better understanding of the interaction between EV of the parasite and uninfected host cells in order to prepare these cells for invasion and/or modulate the host's responses to *T. cruzi*. Knowledge concerning the pathogenic processes and virulence factors secreted by the parasite is needed for the design of prophylactic and therapeutic approaches for Chagas disease.

## Results

### Isolation and characterization of extracellular vesicles of trypomastigotes of *T. cruzi*

A protocol that includes differential centrifugation, coupled with a filtration process through 0.22 µm pore filters and ultracentrifugation, was employed for the isolation of EV of trypomastigotes of *T. cruzi* Pan4 strain. The success of this protocol was evaluated using transmission electron microscopy, atomic force microscopy and nanoparticle tracking analysis. Nanoparticle tracking analysis revealed EV with a mean size of 94 nm ± 57 nm and a mode of 39 nm, results that coincide with the reported size of EV secreted by trypomastigotes of *T. cruzi* previously described by our group^14^.

**Figure 1:**
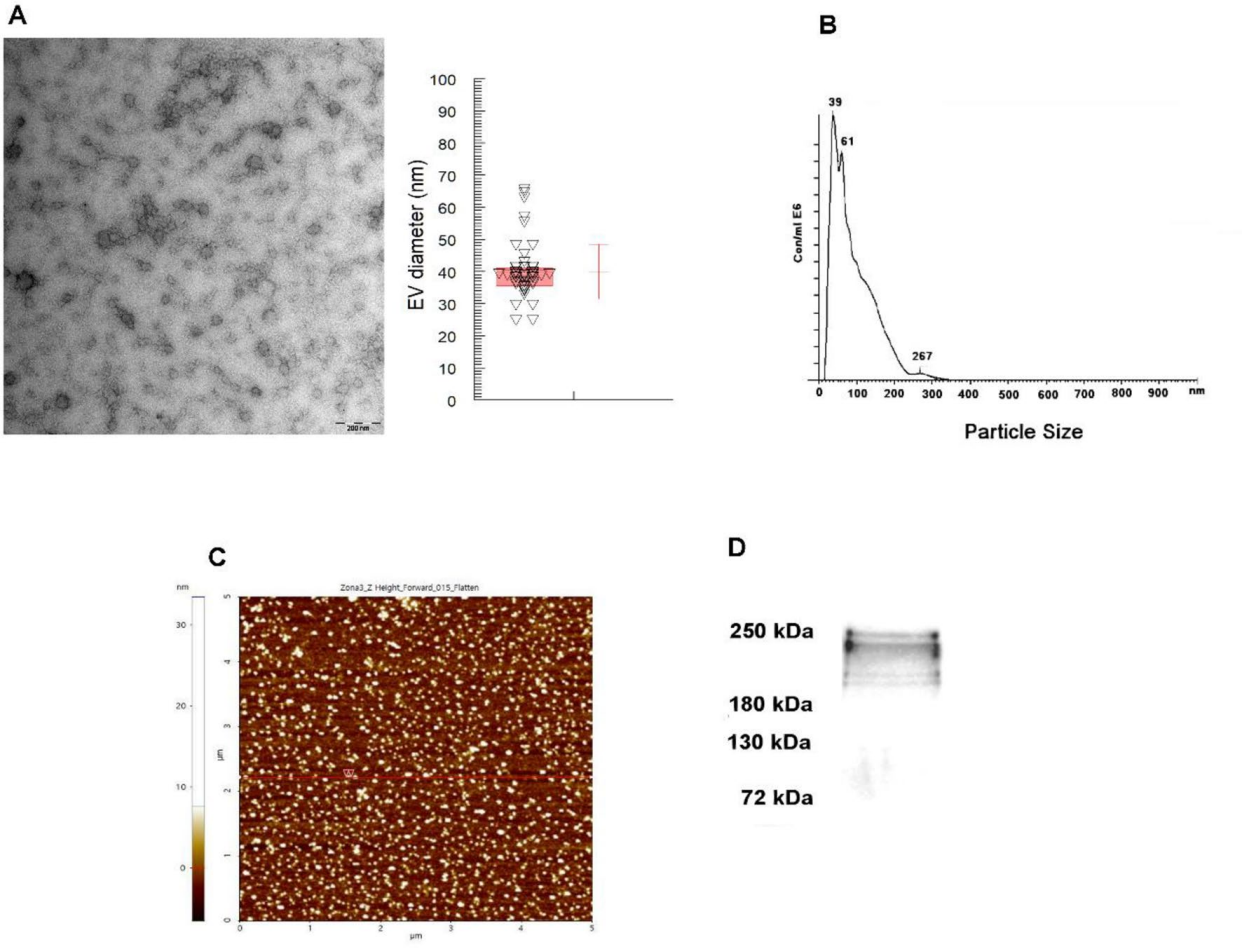
Isolation of extracellular vesicles of trypomastigotes of *T. cruzi* Pan4 strain: (**A**) transmission electron microscopy image of EV (scale bar: 200 nm, mean size: 39.9 nm, mode: 38.8 nm); (**B**) nanoparticle tracking analysis size distribution of EV (mean size: 94 nm ± 57 nm, the largest peak of number of particles corresponding to a size of 39 nm); (**C**) atomic force microscopy image of EV and, (**D**) Western blot analysis for the confirmation of *trans*-sialidase in EV of trypomastigotes.

Western blot confirmed the presence of *trans*-sialidase in EV of this stage of the parasite. Results of the characterization analyses of the EV of trypomastigotes are summarized in Fig. 1.

### Effect of EV of trypomastigotes of *T. cruzi* over Vero cell cultures

Results from the transcriptome analysis revealed 322 differentially expressed genes (DEGs); among those, 168 genes were upregulated (52%, LogFC > 2) and 154 genes were downregulated (47.6%, < − 2) in cells incubated with EV of the parasite. Representative transcripts with the highest fold change differences are displayed in Table 1.

**Table 1:** Differentially regulated transcripts (identified by their accession numbers and gene names IDs) with the highest fold change (LogFC) from the comparison of cells incubated with EV of trypomastigotes of *T. cruzi* (CEV) to control cells (CC).

Accession number	Gene ID	CC	CEV	LogFC	Prob	Biotype	Name
NR_122069	HOXA-AS2	18.83	0.50	5.23	0.98	lncRNA	HOXA cluster antisense RNA 2
XM_024451477	CCDC106	17.65	0.50	5.14	0.98	protein_coding	Coiled-coil domain containing 106 (CCDC106)
NM_001330096	NRXN1	16.48	0.50	5.04	0.98	protein_coding	Neurexin 1
XM_017011231	TCP10	16.48	0.50	5.04	0.98	protein_coding	t-complex 10
XR_929124	LOC105375935	14.12	0.50	4.82	0.97	lncRNA	Uncharacterized LOC105375935
NR_104037	UBE2C	23.54	0.87	4.76	0.99	protein_coding	Ubiquitin conjugating enzyme E2 C
NM_001014985	GLTPD2	12.94	0.50	4.69	0.97	protein_coding	Glycolipid transfer protein domain containing 2
NM_007200	AKAP13	12.94	0.50	4.69	0.97	protein_coding	A-kinase anchoring protein 13
NM_015055	SWAP70	12.94	0.50	4.69	0.97	protein_coding	Switching B cell complex subunit SWAP70
NR_123740	RIMKLB	12.94	0.50	4.69	0.97	protein_coding	Ribosomal modification protein rimK like family member B
NM_024093	C2orf49	0.50	13.90	− 4.80	0.97	protein_coding	Chromosome 2 open reading frame 49
XR_002956178	PARP8	0.50	13.90	− 4.80	0.97	protein_coding	Poly(ADP-ribose) polymerase family member 8
NM_207404	ZNF662	0.50	14.77	− 4.88	0.97	protein_coding	Zinc finger protein 662
NM_001184763	UHMK1	0.50	15.64	− 4.97	0.98	protein_coding	U2AF homology Motif kinase 1
XR_946261	LOC101927445	0.50	15.64	− 4.97	0.98	lncRNA	uncharacterized LOC101927445
NM_001008216	GALE	0.50	17.38	− 5.12	0.98	protein_coding	UDP-galactose-4-Epimerase
XR_944683	SMARCD1	0.50	17.38	− 5.12	0.98	protein_coding	SWI/SNF related
XR_001749246	LOC105369890	0.50	19.11	− 5.26	0.98	lncRNA	Uncharacterized LOC105369890
NR_146113	VPS8	0.50	19.98	− 5.32	0.98	protein_coding	VPS8 subunit of CORVET complex
XM_017026805	LOC390877	0.50	19.98	− 5.32	0.98	protein_coding	Adenylate kinase isoenzyme 1-like

Initial enrichment representation analyses were performed using the set of highly modulated DEGs (separating upregulated and downregulated list of genes), against the GO/KEGG databases by DAVID (Database for Annotation, Visualization and Integrated Discovery (DAVID)) and ClusterProfiler, as well as publicly available gene sets. Most of the detected GO terms and KEGG pathways displayed relevant *p* values but failed to pass an acceptable FDR cut-off, likely due to the small sample size (Table S1).

**Figure 2:**
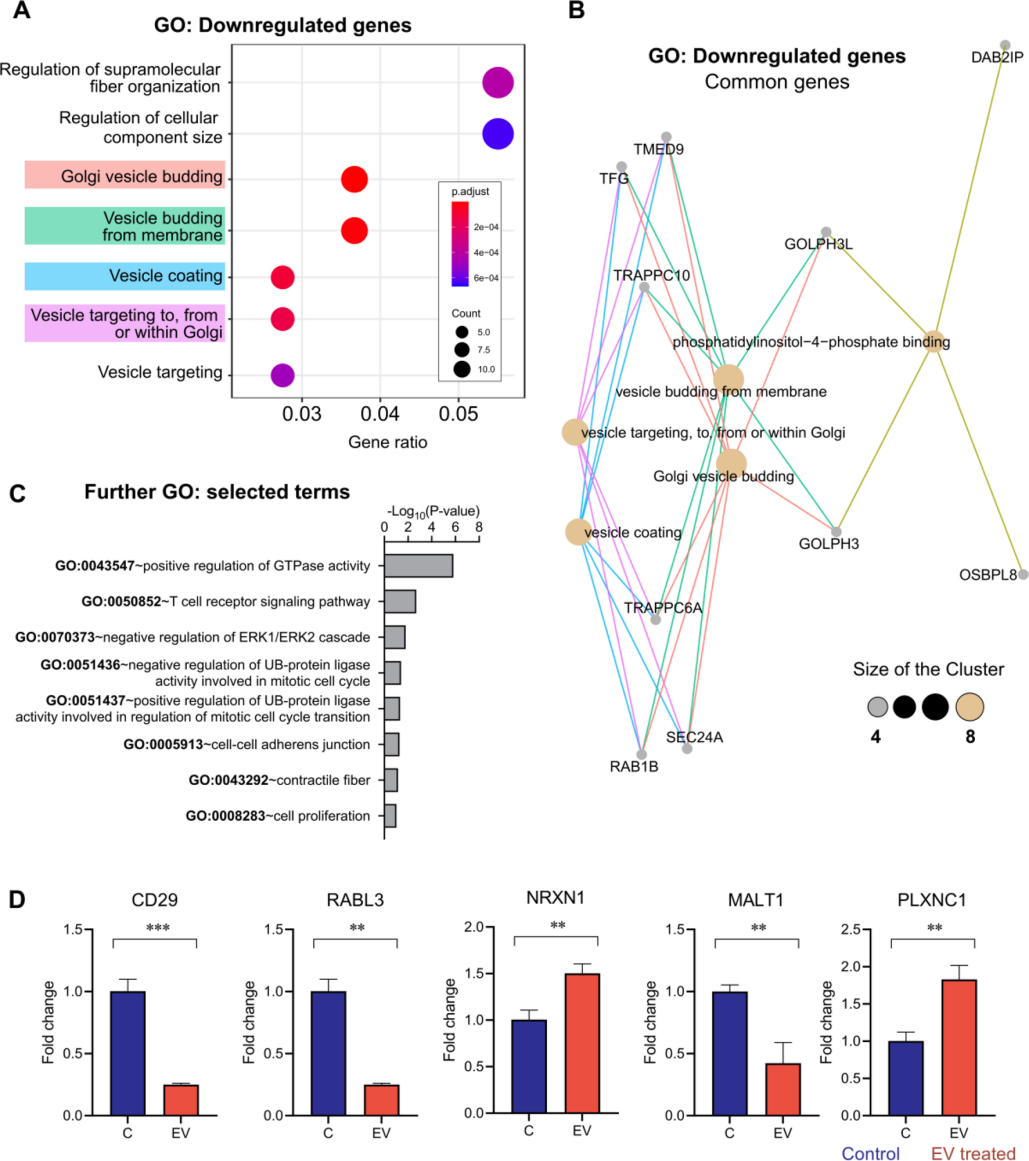
Transcriptomics analysis of cells incubated with extracellular vesicles of trypomastigotes of *T. cruzi.* Enrichment analyses suggest that EV of trypomastigotes downregulate genes that belong to processes related to vesicle formation, deubiquitylation/SUMOylation, as well as cell cycle control. (**A**) Top-ranked GO processes detected by enrichment analyses on downregulated DEGs, followed by a topological representation of the common genes in the top four ranked processes (**B**), as determined by ClusterProfiler. (**C**) Selected GO processes detected by enrichment analyses using the highly modulated DEGs, as determined against the DAVID database and (**D**) respective relevant genes validated by RT-qPCR. Gene expression was calculated as fold induction caused by the respective treatment/control as compared to the expression of *GAPDH*. Data are presented as shown above, with mean ± S.D. (n = 3). Differences to this value were analyzed by one sample Tukey multiple comparison test signed rank tests (asterisks), where ** *p* < 0.01; *** p < 0.001.

In terms of gene ontology (GO), a large enrichment of terms related to vesicle forming/processing biological processes was observed among the downregulated transcripts (*p* value ≤ 0.01, Fig. 2A). These GO processes can, however, be summarized by the downregulation of a few common genes (Fig. 2B), such as *TrappC6A* (LogFC = − 3.26), *TrappC10* (LogFC = − 4.60), *TMED9* (LogFC = − 3.47), *TFG* (LogFC = − 4.11) and *Rab1b* (LogFC = − 2.05), as well as *Sec24A* (LogFC = 2.76), which showed only a marginal modulation when re-validated using RT-qPCR.

**Figure 3:**
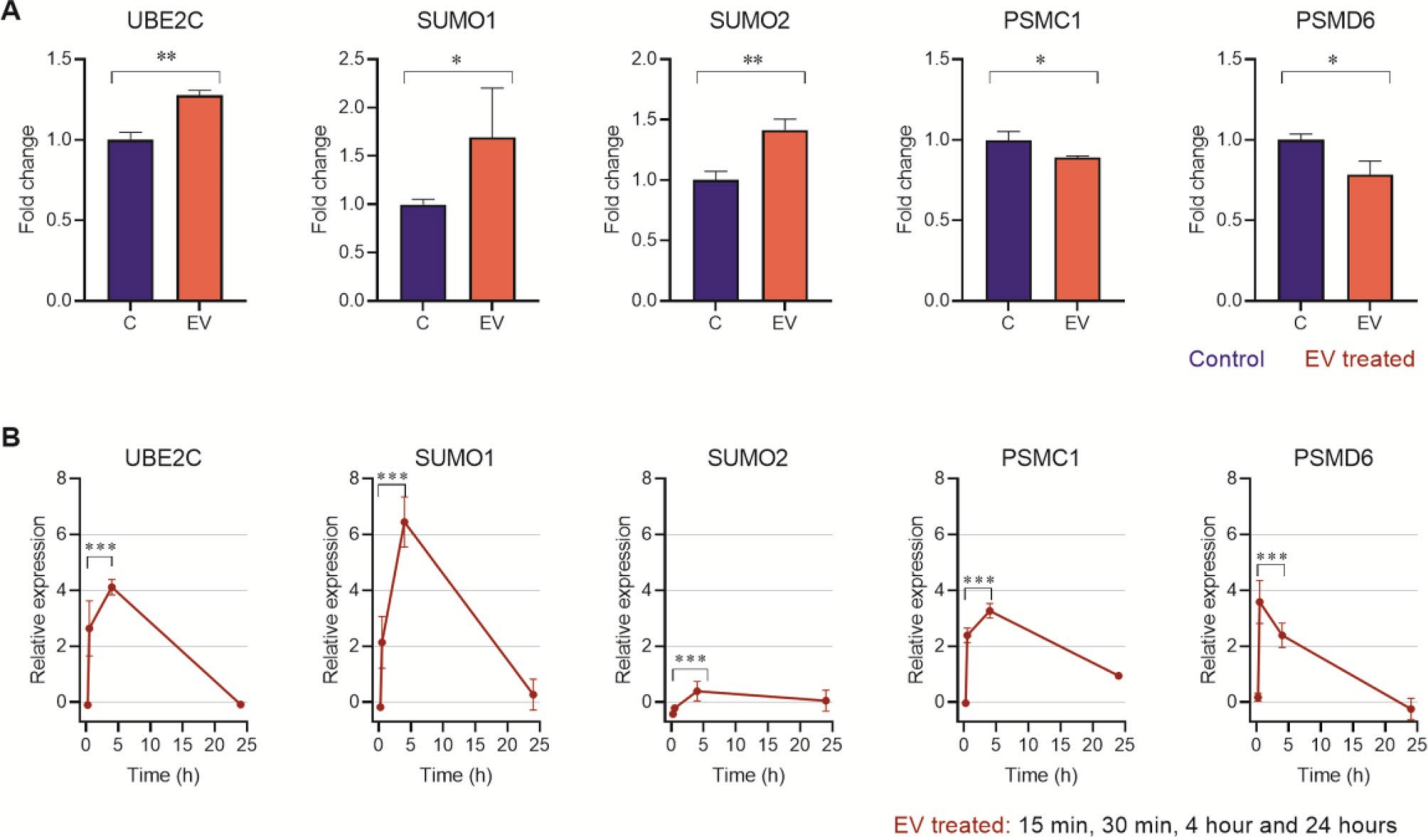
Expression analysis of genes involved in SUMOylation in Vero cells incubated with extracellular vesicles for 4 h (**A**) and cell cultures of Vero cells incubated with cell derived trypomastigotes of *T. cruzi* at different times during the process of infection (**B**). Gene expression was calculated as fold induction caused by the respective treatment/control as compared to the expression of *GAPDH*. Data are presented as shown above, with mean ± S.D. (n = 3). Differences to this value were analyzed by one sample Tukey multiple comparison test signed rank tests (asterisks), where * *p* < 0.05; ** *p* < 0.01; *** *p* < 0.001.

GO terms such as T-cell receptor signaling pathway and ubiquitin/SUMO appeared as relevant among the downregulated pathways, which not only agree with gene set enrichment analyses (Fig. S1A, B), but also contain some of the lowest ranked DEGs in terms of expression levels. Ubiquitin-related processes represented by the ubiquitin ligase components *Ube2C* (LogFC = 4.76), as well as supporting genes such as *SUMO1* and *SUMO2*, were shown to be upregulated in the RT-qPCR validation (Fig. 3), while other components such as *Uba52* (LogFC = − 3.15), *PSMD6* (LogFC = − 3.26) and *PSMC1* (LogFC = − 4.32) were downregulated.

In Fig. 3A, an overexpression of *UBE2C*, *SUMO1* and *SUMO2* in cells incubated with EVs of trypomastigotes is observed, which coincides with expression results of cells incubated with trypomastigotes for the same time-point (Fig. 3B). Figure 3B also shows the result of a control experiment where the expression levels of *SUMO1, SUMO2, UBE2C, PSMC1* and *PSMD6* genes analyzed by RT-qPCR in cells undergoing parasitization by the infective trypomastigote stage of the parasite. In this case, a downregulation in the expression of *SUMO1, SUMO2, UBE2C* and *PSMC1* was observed 15 min post-interaction when compared to the expression levels of the uninfected control cells. Subsequently, after 30 min of incubation, all genes except for *SUMO2* were upregulated. The same phenomenon was observed 4 h post-interaction, including at that time-point the increased expression of *SUMO2*. However, expression levels of all genes decreased 24 h post-interaction, showing that this pathway was no longer relevant in this time point and motivating us to skip it in the next analyzed gene sets.

#### Expression of RhoA, Rac1 and Cdc 42

**Figure 4:**
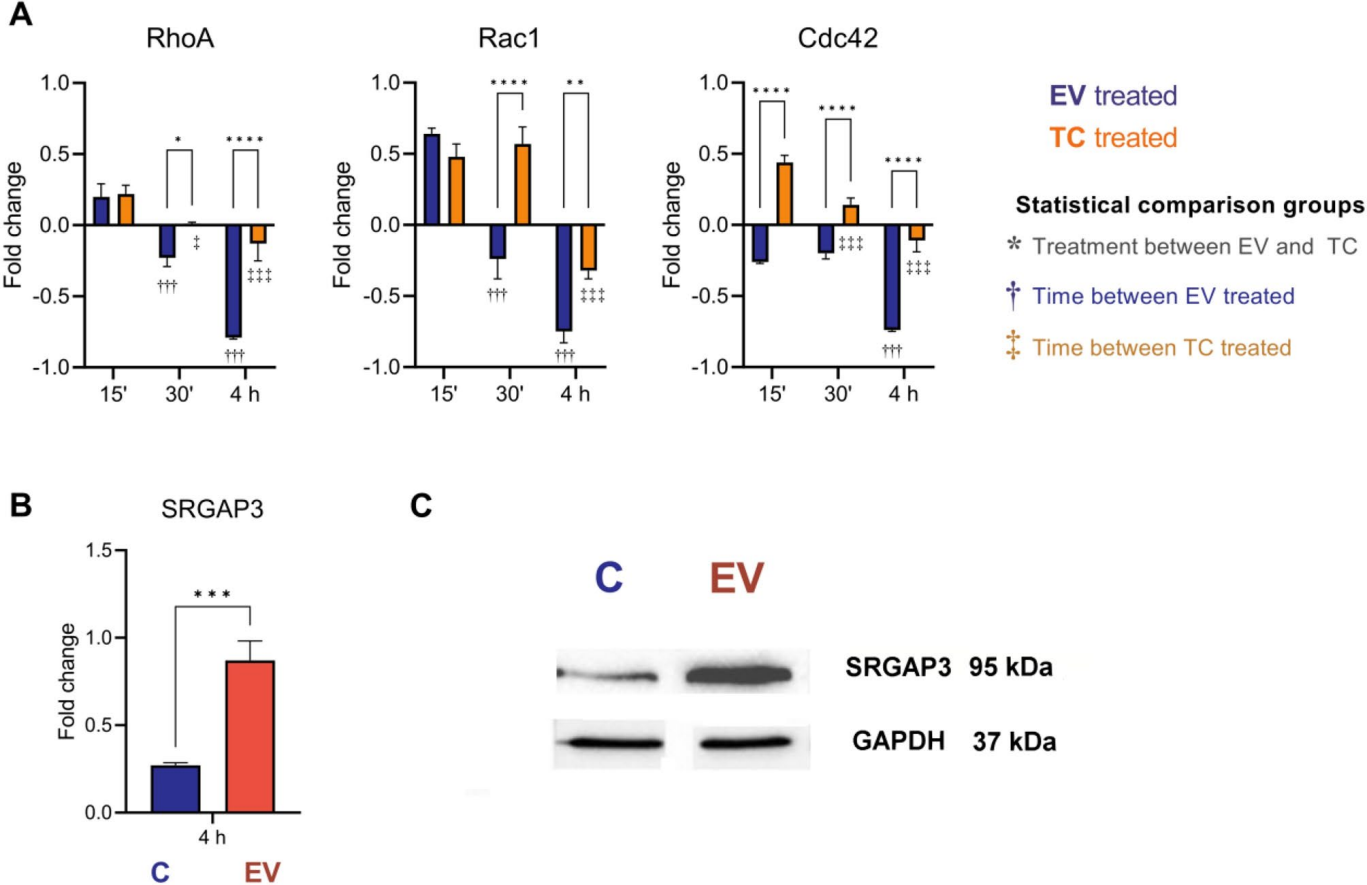
Changes in the expression of Rho-GTPase genes (*RhoA*, *Rac1* and *Cdc42*) induced by extracellular vesicles secreted by trypomastigotes of *T. cruzi*. (**A**) An overexpression of *RhoA* and *Rac1* was observed 15 min post-incubation of Vero cells with EV, while the expression of *Cdc42* was downregulated. After this time- point, a downregulation in the expression of the 3 analyzed genes appeared at 30 min, being more evident 4 h post-interaction. The incubation of cells with trypomastigotes revealed an overexpression of the 3 genes both 15 and 30 min post-interaction, while the same behavior of EV in downregulating the expression of the 3 genes was observed 24 h post-interaction. Gene expression was calculated as fold induction caused by the respective treatment/control as compared with the expression of *GAPDH*. Data are presented as shown above, with mean ± S.D. (n = 3). Differences to this value were analyzed by one-way ANOVA using the Tukey’s post correction as rank tests (asterisks), where * *p* < 0.05; ** *p* < 0.01; **** *p* < 0.0001, comparing within the same time stamp the trypomastigotes (TC, orange) and the EV controls (blue). Comparison of each time point against the 15 min time are represented as †, for the TC groups, and ‡ for the EV groups. Expression levels of SRGAP3, an activating protein of Rho-GTPases, in cells incubated with EV of trypomastigotes of *T. cruzi* for 4 h, derived from the mRNA levels (**B**) and on protein levels (**C**). Expression was calculated as fold induction caused by the respective treatment/control as compared with the expression of GAPDH. Data are presented as shown above, with mean ± S.D. (n = 3). Differences to this value were analyzed by one sample Tukey multiple comparison test signed rank tests (asterisks), where *** *p* < 0.001.

Figure 4 shows the changes in the expression levels of *RhoA*, *Rac1* and *Cdc42* after the incubation of cells with EV trypomastigotes of *T. cruzi.* The overexpression of *RhoA* and *Rac1* is observed after 15 min of incubation, while the expression of *Cdc42* is downregulated (Fig. 4). After this time-point, a downregulation in the expression of the 3 genes appeared at 30 min, being more evident at 4 h of the EV-cells interaction (Fig. 4). Moreover, the incubation of trypomastigotes of the parasite with cells showed overexpression of the 3 genes both 15 and 30 min post-incubation, while at 4 h the same behavior as EV in downregulating the expression of the 3 genes was observed 4 h post-interaction.

#### Effect of EV of *T. cruzi* over apoptosis

**Figure 5:**
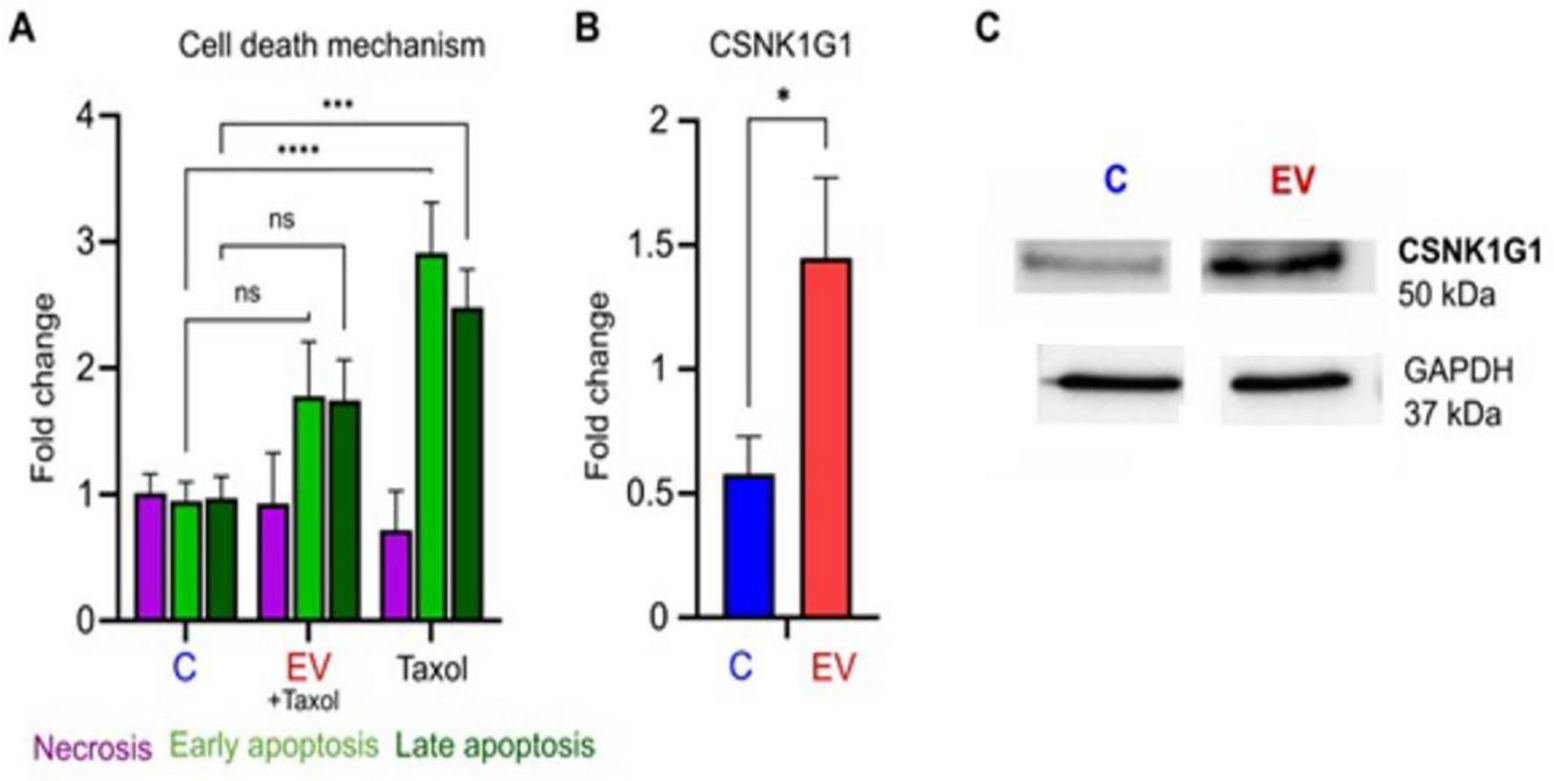
“Protective” effect of extracellular vesicles of trypomastigotes of *T. cruzi* over apoptosis of Vero cells. (**A**) Significant lower relative values of cells in early and late apoptosis were observed in cells incubated with EV and then treated with taxol for 72 h. The values are represented as the mean percentages ± SEM. Tukey–Kramer test, where *** *p* < 0.001; **** *p* < 0.0001 and ns: non-significant differences. mRNA expression levels (**B**) and Western blot analysis (**C**) of casein kinase protein CSNK1G1 that shows an increase in protein synthesis after the interaction of cells with EV of the parasite. Expression was calculated as fold induction caused by the respective treatment/control as compared with the expression of GAPDH. Data are presented as shown above, with mean ± S.D. (n = 3). Differences to this value were analyzed by one sample Wilcoxon signed rank tests (asterisks), where * p < 0.05.

Flow cytometry was employed to evaluate if EV of trypomastigotes could have an effect on the apoptosis of Vero cells. As shown in Fig. 5, relative values of cells in early and late apoptosis were significantly lower in cells incubated with EV of the parasite than in cells treated only with taxol (Fig. 5A), which could suggest a “protective” role of EV against this type of cell death. Moreover, Western blot expression analysis of casein kinase protein CSNK1G1 revealed an increase in protein synthesis after the interaction of cells with EV of the parasite (Fig. 5B).

## Discussion

Extracellular vesicles of *Trypanosoma cruzi* are one of the most studied vesicles in parasitology, and their roles in affecting cell physiology, functionality and the immune response have been confirmed. However, explanations for these roles at a molecular level are still scarce. To understand the influence of the vesicles of the parasite over cells at this level we performed transcriptome analyses, in which RNAs were obtained from Vero cells incubated for 4 h with EV of cell derived trypomastigotes and libraries were prepared and sequenced in a fraction of a NovaSeq6000 S4 flow cell (Illumina).

Sequencing results revealed that 322 genes were found to be differentially expressed genes (DEGs) in cells incubated with EV of the parasite: 168 genes were upregulated (52%, LogFC > 2) and 154 genes were downregulated (47.6%,  < − 2). Among those with known functions, the upregulation of the long non-coding RNA of HOXA-AS2, whose shRNA was shown to act as an apoptosis repressor^20^ is highlighted and seems to be in accordance with results from the apoptosis assays, which will be discussed below. Moreover, this observation is in line with previous results from the group^19^ in which EV-treated cells were arrested in the G0/G1 cell cycle phase and other physiological alterations were reported, such as permeabilization of cell membranes and the disruption of actin filaments. In this sense, changes in the expression of *RhoA*, *Rac1* and *Cdc42*, Rho-GTPases involved in different cell responses and considered key regulators of the actin cytoskeleton were also observed in cells incubated with EV of the parasite, as well as an overexpression of SRGAP3, a protein that blocks the activity of Cdc42^21^. Altogether, these findings could explain, at least in part, some of the above- mentioned results, as well as previously published results^19^ that motivated transcriptomics analysis included in this work.

On the other extreme, the downregulation of VPS8 expression (LogFC = − 5.32) was identified, which, together with VPS3 (also known as TRAP1), is required for the recycling of β1 integrins^22,23^. Jonker et al. showed that the depletion of either VPS3 or VPS8 delays the delivery of internalised integrins to recycling endosomes and their return to the plasma membrane. This integrin recycling is fundamental for the interaction between the cell and the extracellular matrix as well as in invasion and migration^24^.

The increased expression of both SUMO ligase and its substrates, together with the downregulation of ubiquitin cleaving proteins could be implicated in the deregulation in mitosis controlling proteins, as suggested by their respective GO terms and by observations that SUMOylation/ubquitinylation deregulation leads to cell cycle arrest in mammalian lines, as well as cancer^25^, depending on the cell type. SUMOylation is a key process in a number of cellular, nuclear, metabolic and immunological processes, and consists in a post-translational modification of proteins that regulates protein stabilization, nucleus-cytoplasmic transport and protein–protein interactions. More specifically, SUMOylation involves the modification of one or more lysine residues of target proteins by conjugation of a small ubiquitin-like polypeptide, known as SUMO, for degradation, stability, transcriptional regulation, cellular localization and transport^26^.

Deregulation in SUMOylation and deSUMOylation has been described in intracellular pathogens such as herpes simplex virus-1 (HSV-1)^27^ and influenza virus (types A and B; IAV and IBV)^29^. In the case of HSV-1, a widespread reduction in the levels of cellular SUMOylated species induced by ubiquitin ligase ICP0 was reported; an activity that correlates with relief from intrinsic immunity antiviral defense mechanisms^28^. For influenza, it was shown that the infection leads to a viral replication-dependent global increase in cellular SUMOylation^28^. More recently, a significant decrease in the expression of E3 SUMO ligase RanBP2^29,30^ has been described in SARS CoV2-infected cells. Intracellular bacteria could also modulate the host SUMOylation machinery, so that intracellular development of the pathogen can take place, as is the case in *Shigella flexneri*^31^, *Salmonella typhymurium*^32,33^, *Yersinia*^34^, among others.

In a similar way, it has been shown that the development and intracellular access of parasitic protozoa also modulates the SUMO machinery of the host cell. For example, sporozoites of *Plasmodium berghei* invading mammalian hepatocytes during their exaerythrocytic development can induce modifications in the expression of SUMOylation enzymes, which are significantly downregulated^35^. An inhibition of host cell SUMOylation was also observed in cells during the process of *Toxoplasma gondii* infection^35^. In trypanosomatids, it has been reported how the host SUMOylation pathway negatively regulates protective immune responses, which promotes the survival of *Leishmania donovani*^26^. Results from the study by Singhal et al. revealed that the infection of macrophages with the promastigote stage of this parasite leads to upregulation of SUMOylation pathway genes and downregulation of the SUMOylation gene *SENP1*, while infection with the amastigote stage leads to downregulation of proteins involved in SUMOylation^26^. In our experiments, *SUMO1* and *SUMO2*, as well as *UBE2C*, were upregulated in the transcriptome analysis of cells incubated with EV with respect to control cells, while a downregulation of *PSMD6* was observed in cells incubated with these vesicles. However, results from control experiments using trypomastigotes of *T. cruzi* revealed a substantial drop in the expression levels of *SUMO1*, *SUMO2*, *UBE2C* and *PSMC1* 15 min after the trypomastigote-cell interaction with respect to uninfected control cells, although *PSMD6* showed a slightly higher expression (Fig. 3B). This specific time-point corresponds to the time when the parasite contacts the cell membranes to initiate the invasion process. Subsequently, at 30 min, the expression levels for all the analyzed genes involved in SUMOylation were upregulated (except for *SUMO2*), and the same phenomenon was observed 4 h after the infection. After 24 h of infection, the expression levels of *UBE2C* and *PSMD6* the expression levels returned to unstimulated baseline levels, while *SUMO1, SUMO2* and *PSMC1* showed slightly higher expression levels than uninfected control cells.

During the process of *T. cruzi* invasion of mammalian host cells, both cell culture-derived trypomastigotes ("blood" trypomastigotes and metacyclic trypomastigote forms) attach to the cell membrane using an array of different surface proteins. The parasite then invades the cell, leading to the formation of the parasitophorous vacuole to which the lysosomes fuse and both infecting forms induce similar cellular modifications including an increase in cytoplasmic calcium^36^. Lysosomal acidification is an important signal for activating key mechanisms that allow the parasite escaping from the parasitophorous vacuole into the cytoplasm, where it differentiates into the replicative amastigote stage and multiplies actively^5,36,37^. In contrast, promastigotes of *Leishmania* must be phagocytosed by neutrophils and macrophages and establish an intracellular residence inside the phagolysosome, where they transform into amastigotes and multiply^38^. The mechanism of inhibition of the initial expression of SUMO proteins of the cell by trypomastigotes could be considered a survival mechanism while the parasite is located inside the parasitophorous vacuole prior to its escape to the cytosol. Although EV of the parasite are able to modify some physiological aspects of the host cell, modulation of the lysosomal response in the early stages of EV-cell interaction has not yet been demonstrated, contrary to what occurs after the parasite´s entry, which would explain the differences in the SUMOylation of cells.

Changes in gene expression by the incubation of cells with EV of *T. cruzi* have been previously published by Garcia Silva et al. (2014), which employed a different cell line (HeLa), as well as EV secreted by the non-infective stage (epimastigotes) of the parasite for the vertebrate host, and a different strain of the parasite (DM28c)^39,40^. In their studies, the authors revealed changes in the expression of different genes, and the elicited responses modified mainly the host cell cytoskeleton, the extracellular matrix and immune response pathways and described gene expression changes in Rho-GTPases, IL-1, IL-1R, IL-6, IL-18, among others^39^. For these authors, some of the affected pathways were of particular interest in light of the infection-favoring role of EV of *T. cruzi*, such as the Rho-GTPase signaling pathway that was affected both at 6 and 24 h post-incubation with the EV; in this sense, genes belonging to this pathway were modified in a way that would keep this signaling pathway inactive^39^. As previously mentioned, this family of proteins regulates aspects related to motility and cell morphology through the rearrangement of the cytoskeleton and the regulation of the actin cytoskeleton is one of the pathways identified as affected by the incubation with EV^19^. As stated by Garcia Silva et al. cytoskeletal reorganization is recognized as one of the main processes that takes place during the entry of *T. cruzi*, with actin depolymerization most likely facilitating this entry^39^. In this context, EV-triggered depolymerization of the actin cytoskeleton in the early stages of the interaction would facilitate the initial entry of *T. cruzi* into the host cell. Indeed, EV affect host cell actin filaments, which allows the migration of lysosomes and the formation of the parasitophorous vacuole required for parasite internalization^41^.

In this study, results from our experiments revealed downregulation of *Cdc42* and overexpression of *RhoA* and *Rac1* 15 min after the interaction of cells with EV, in contrast to the interaction of cells with the infective trypomastigote stage of the parasite; however, a downregulation of the 3 genes 30 min and 4 h after the interaction of cells with EV was then observed, a phenomenon that coincides with results obtained at 4 h post-interaction. As previously mentioned, the overexpression of the protein SRGAP3 after the 4-h EV-cell interaction is noteworthy, as it is a protein that blocks Cdc42^21^ in particular. Besides, increased concentrations of intracellular free calcium and disruption of the actin filaments could be also related to the effect EV of trypomastigotes exert over Rho-GTPases signaling pathways.

EV have gained considerable interest as mediators of cell death. In this sense, the results presented in this work (Fig. 5) suggest a “protective” role of EV of *T. cruzi* against apoptosis, as relative values of early and late apoptosis of cells incubated with EV and then treated with taxol at a low concentration are significantly lower than cells treated only with the anti-neoplasic agent. Taxol (paclitaxel) acts by promoting tubulin dimerization and inhibiting the depolymerization of microtubules, which results in the formation of abnormally stable and non-functional microtubules^42^; as a result of continuous exposure, mitosis could not be completed, resulting in mitotic metaphase arrest and cellular toxicity^43,44^.

The anti-apoptotic role of EV of other origin has been demonstrated by several authors^45–49^. In the case of EV secreted by microorganisms, both roles of the vesicles in attenuating or promoting cell death^50^, depending on different factors, including the species, have been demonstrated. In the specific context of parasites, it has been reported that EV secreted by the protozoan parasites *Toxoplasma gondii* and *Leishmania* can induce in vivo a proinflammatory response and apoptosis^50,51^. Besides, it has been reported that the EV of *Plasmodium falciparum* are able to induce programmed cell death processes in the parasite`s population by inducing apoptosis in highly parasitized cultures^52^. For *T. cruzi*, this is the first study that evaluates the role of EV in apoptosis. It is noteworthy that transcriptomic analysis supports this possible anti-apoptotic role as the upregulation of the long non-coding RNA of HOXA-AS2 was found, mentioned above. Besides, overexpression of CSNK1G1, a protein of the casein kinase family, was identified by Western blot. Isoforms of the casein kinase 1 (CK1) family have been shown to phosphorylate key regulatory molecules involved in the cell cycle, transcription and translation, the structure of the cytoskeleton, cell–cell adhesion and receptor-coupled signal transduction; moreover, they regulate key signaling pathways known to be critically involved in tumor progression^53^. Although most evidence points to important regulatory roles of the isoforms CK1α, CK1δ and CK1ϵ, and the role of the gamma-isoforms is still enigmatic and not very well investigated^53^ an anti-apoptotic function of CK1α in the extrinsic apoptosis pathway has been shown. As the isoforms have a high homology^53^, we could also hypothesize an anti-apoptotic role of CSNK1G1. However, more studies are needed to confirm this hypothesis and to describe in detail the apoptotic signaling pathways that EV of *T. cruzi* exert.

It has been reported that shed trypomastigote components present in parasite-conditioned medium are implicated in triggering several signaling cascades in host cells to facilitate different processes, including the activation of anti-apoptotic responses^54^; more specifically, *trans*-sialidase purified from conditioned supernatants of Vero cells infected with the *T. cruzi* Silvio-X10/4 strain was able to induce neurite outgrowth and rescued PC12 cells from apoptotic death caused by growth factor deprivation^55^. The presence of different *trans*-sialidases in the proteome of EV of *T. cruzi* has been confirmed in previous works by our research group^13^. There are also several lines of evidence that *T. cruzi* can up- or downregulate apoptosis of fibroblasts and macrophages, and that the role of apoptosis in the pathogenesis of the disease is related to the parasite clones and their different abilities to invade and proliferate within host cells^56^. In this regard, the anti-apoptotic effect of EV observed in this study could vary employing other cell lines and strains of the parasite. Therefore, the effect of EV of *T. cruzi* over apoptosis ought to be investigated in more detail, as EV carry a variety of proteins and microRNAs into target cells and cell apoptosis is a complex multi-pathway process.

## Methods

### Cell culture, parasite strain and infection of cells with *T.* cruzi

Vero cells (ECACC 84113001) were cultured in Nunc cell-culture flasks of 75 cm^2^ surface area (Thermo Fischer Scientific, USA) with Modified Eagle’s Medium (MEM) (Sigma, USA) supplemented with 10% inactivated fetal bovine serum (iFBS) (Gibco, USA) plus antibiotics (penicillin 100 U/mL, streptomycin 100 μg/mL). The cell cultures were maintained at 37 °C, in a moist atmosphere enriched with 5% CO_2_.

Cells were initially infected with purified metacyclic trypomastigotes of the Pan4 (Tc Ia + Tc Id) strain of *T. cruzi*, isolated in Panama in 2004 and maintained in culture and cryopreserved in our laboratory. Vero cell monolayer was disrupted with trypsin–EDTA solution, the cells were counted in a Neubauer chamber and placed on a new culture flask at 1 × 10^6^ cells/mL in MEM + 10% iFBS. Once the cells were attached to the surface of the flask (approximately after 12 h), cells were washed with Hank's solution to remove iFBS and infected with a suspension of the metacyclic forms obtained in culture and purified in Percoll according to the methodology described by Castanys et al.^57^. Cell cultures were incubated with the parasites for 6 h in MEM without iFBS, the parasite/cell ratio was 3 parasites per cell. After this time, parasites present in the cell culture supernatant were removed, cell cultures were washed with Hank`s solution and fresh MEM + 10% iFBS was added. After 120 h of the intracellular development of the parasite, tissue-culture cell-derived trypomastigotes (TcT) were harvested by centrifugation^58^. For this purpose, the culture medium was collected from the infected monolayers every 24 h and then centrifuged at 3000 × g for 5 min. The resulting pellet with the parasites was washed in sterile PBS four times and resuspended in RPMI culture medium (Sigma, USA) buffered with 25 mM HEPES (pH 7.2) and supplemented with 10% exosome-free iFBS, for the subsequent isolation of EV.

The cell line was routinely monitored for *Mycoplasma* by PCR, using primers GPO-3 and MGSO as described by van Kuppeveld et al. in 1994^59^.

### Isolation of extracellular vesicles

EV of trypomastigotes of *T. cruzi* were obtained as previously described by Díaz Lozano et al. and Retana Moreira et al.^19,60^. Briefly, 5 × 10^7^ trypomastigotes of *T. cruzi* were incubated for 5 h in 5 mL RPMI 1640 medium (Sigma Aldrich, St. Louis, MO, USA) buffered with HEPES 25 mM (pH 7.2) and supplemented with 10% exosome-free heat inactivated fetal bovine serum (iFBS). After this time, the parasites and cell debris were eliminated after centrifugation at 3500 × g for 15 min and the supernatant was collected. In order to eliminate larger EV and to obtain an exosome-enriched pellet, the supernatant was centrifuged at 17,000 × g for 30 min at 4 °C and then filtered through a 0.22 µm-pore filter (Sartorius, Göttingen, Germany), for subsequent ultracentrifugation at 100,000 × g for 18 h in an Avanti J-301 ultracentrifuge (Beckman Coulter, Brea, CA, USA) with a JA-30.50 Ti Rotor. In this case, the resulting pellet was washed three times in sterile-filtered (0.22 µm pore filter) PBS, using a Sorval WX80 ultracentrifuge (Thermo Fisher Scientific, Waltham, MA, USA) with a F50L-24 × 1.5 fixed-angle rotor, and finally resuspended in 100 µL PBS. The viability of trypomastigotes after the 5-h period of EV secretion was evaluated using the trypan blue exclusion test, maintaining a percentage of 99% viable parasites.

The EV isolation procedure was evaluated by transmission electron microscopy, atomic force microscopy and nanoparticle tracking analysis, as described in previous works^13^. The protein concentration of each EV was quantified using the Micro-BCA protein assay (Thermo Fischer Scientific, Waltham, MA, USA) and the presence of *T. cruzi-*specific protein markers in EV was evaluated by Western blot.

### Transmission electron microscopy

To confirm the production of EV by trypomastigotes of *T. cruzi*, EV obtained after the isolation procedure were resuspended in 30 µL sterile-filtered PBS (pH 7.3) and 5 µL of the suspension was adsorbed directly onto Formvar/carbon-coated grids. After 30 min, the grids were washed in PBS, fixed in 1% glutaraldehyde for 30 min, washed once again in PBS and stained and contrasted with 2% (v/v) uranyl acetate. The visualization of the samples was achieved using a Carl Zeiss LIBRA 120 PLUS SMT electron microscope at the “Centro de Instrumentación Científica” of the University of Granada, and the size of the vesicles was measured using the Image J 1.41 software.

### Atomic force microscopy

Topographic imaging of the EV of *T. cruzi* was performed using the non-contact AFM mode in an NX-20 instrument (Park Systems, Suwon, Korea) with ACTA cantilevers (K = 40 N m^–1^ and f = 320 kHz), as previously described^13^. Briefly, each EV sample was diluted 1:4 in sterile-filtered PBS and 8 µL of the dilution of EV was deposited onto freshly cleaved muscovite mica. After 10 min, the substrate with the sample was rinsed three times with MilliQ water (Millipore, Burlington, MA, USA) and further dried with a gentle stream of argon. Images were typically acquired as 256 × 256 pixels at a scan rate of 0.5–0.7 Hz, and processed and analyzed using XEI software (Park Systems, Suwon, Korea). Representative images of samples were obtained by scanning at least 3 different locations on at least 3 different samples.

### Nanoparticle tracking analysis

Distribution, size, and concentration of the EV samples of *T. cruzi* were determined by measuring the Brownian motion rate according to the particle size using a NanoSight NS300 (Malvern Instruments, UK), a system equipped with an sCMOS camera and a blue 488 nm laser beam. For the analysis, samples of EV were diluted 1/100 in low-binding Eppendorf tubes with sterile-filtered PBS just before the measurements, which were performed at 25 °C. For data acquisition and information processing, the NTA software 3.2 Dev Build 3.2.16 was used. The particle movement was analyzed by NTA software with the camera level at 16, slider shutter at 1200 and slider gain at 146. The mean size distribution was calculated as a mean of three independent size distributions.

### Identification or protein markers in EV of *T. cruzi* by Western blot

To confirm the presence of *T. cruzi*-specific proteins in EV secreted by trypomastigotes, Western blots using anti-*trans*-sialidase (mAb39) antibody were performed, following the methodology described by Retana Moreira et al. in 2021^13^. For the analysis, 30 µg of EV of trypomastigotes were resolved by SDS-PAGE, transferred to PVDF membranes (Trans-Blot Turbo Midi PVDF Transfer Packs, Bio-Rad Laboratories, USA) for 40 min at 40 V in a Trans-Blot Turbo Transfer System (Bio-Rad Laboratories, USA), and blocked overnight with 5% non-fat milk in PBS-0.1% Tween 20. The membranes were then washed in PBS-0.1% Tween 20 and incubated overnight at 4 °C with the primary antibody anti-TS mAb 39 (1:1000) (produced in mice). After the incubation, the membranes were washed in PBS-0.1% Tween 20 and incubated for 1 h with secondary antibody goat anti-mouse IgGs conjugated with peroxidase (1:1000) (Agilent Technologies, Santa Clara, CA, USA). The reaction was visualized using Clarity ECL Western substrate (BioRad, Hercules, CA, USA) in a ChemiDoc Imaging System (BioRad, Hercules, CA, USA).

### Incubation of Vero cell cultures with EV of *T. cruzi*

Cultures of 1X10^6^ Vero cells were grown for 24–48 h in Nunc 6-well plates (Thermo Fischer Scientific, USA) using MEM supplemented with 10% iFBS and antibiotics, as previously described. Thereafter, the monolayers were washed 3 times in serum-free MEM and incubated for 4 h with EV of trypomastigotes of *T. cruzi* resuspended in serum-free MEM*.* The concentration of total protein of EV employed was 0.38 µg/mL (equivalent to ~ 2.7 × 10^8^ EV/mL), since it was established as the effective dose (ED) 50 in previous investigations by our group, using the same strain of the parasite, EV isolation procedure and incubation conditions^19^. After the incubations, culture media were removed and the cells were submitted to RNA extractions for gene expression analyses by RT-qPCR and transcriptome analysis.

### RNA extraction

Total RNA of the cell cultures incubated with EV of trypomastigotes of *T. cruzi* was extracted using TriZOL reagent (Thermo Fisher Scientific, USA), following the manufacturer’s recommendations. An approximate quantification and purity evaluation of the samples was achieved using a Nanodrop spectrophotometer, after determining absorbance at 260 nm. RNA samples were also quantified and quality-checked in an Agilent 2100 Bioanalyzer, using the Agilent RNA 6000 Kit.

### Transcriptome analysis

In order to determine the effect of EV of trypomastigotes of *T. cruzi* over cells at the transcriptome level, Illumina’s TruSeq Stranded mRNA Library Prep Kit was employed to prepare the libraries, following the manufacturer's instructions. Briefly, each sample was enriched in mRNA by selecting those molecules with a poly-A tail at their 3' end. Captured mRNAs were then converted into cDNA and sequencing adaptors were added to their ends. The samples were dual-indexed for post-sequencing demultiplexing.

The fragment size distribution and concentration of the libraries were checked in the Agilent 2100 Bioanalyzer using the Agilent DNA 1000 Kit and the quantification of the libraries was performed using the Qubit dsDNA HS Assay Kit (Thermo Fisher Scientific, USA). Then, they were pooled in equimolar amounts according to the Qubit results. Finally, the resulting pool was sequenced in a fraction of a NovaSeq6000 S4 flow cell (Illumina).

The quality of the raw sequencing data was checked using FastQC 0.11.15. Reads that contained adapter sequences and/or that showed low-quality were identified and trimmed using Trimmomatic 0.36^61^. Then, a second quality check was performed using FastQC 0.11.15 to make sure only high-quality reads were used for the mapping step.

Read counts were quantified using htseq-counts. The counts were also normalized and filtered following the Trimmed Mean of M-values (TMM) method^62^. Finally, differential expression between the samples (cell control: CC and cells incubated with EV of *T. cruzi*: CEV) were analyzed using the Bioconductor package NOISeq. The probability threshold was set at q = 0.95.

### Bioinformatic analyses

For transcriptome results, differential expression analyses were performed using the edge R package and an experimental based design matrix comparing the EV-incubated samples against the control group (cells without the incubation with EV of *T. cruzi*). Further, differentially expressed genes were identified at the *p* value of < 0.01 and log_2_ fold change > 2 (LogFC).

Integrative analysis was performed in R (v3.6), to obtain the lists of genes that overlap with publicly available datasets of interest. Briefly, a list of differentially expressed genes up or downregulated (DEGs) underwent an unbiased approach to characterize our datasets enrichment analysis of gene ontology terms on our datasets with WebGesalt^63^ and David v6.8 (accessed on April 2021, current version available at https://david.ncifcrf.gov) servers, using both over-representation and gene-set enrichment modes with default settings, as well as in R using ClusterProfiler^64^. Information from all significantly enriched terms (*p* value < 0.05) from all datasets was pooled to interpret the evaluated datasets.

### Gene expression analyses of Rho-GTPases by RT-qPCR

To determine the effect of EV of trypomastigotes of *T. cruzi* over the expression of Rho/Rho kinases in cells, the specific expression of *RhoA*, *Rac1* and *Cdc42* genes was analyzed after the incubation of Vero cells with EV of *T. cruzi* during different time points (15 min, 30 min, 4 h and 24 h). The same analysis was performed after the stimulation with 10^6^ trypomastigotes forms for the different times as positive control. After obtaining the RNA of the cell cultures as described above, reverse transcription was performed using the QuantiTect Reverse Transcription kit (Qiagen, Hilden, Germany) which contains a DNA digestion step. Then, quantitative PCRs were performed with the resulting cDNAs in 10 µL volumes, using a CFX-96 Real Time System (Bio-Rad Laboratories, USA). Each reaction included 5 µL SsoFast® EvaGreen Supermix (Bio-Rad Laboratories, EEUU), 1 µL each forward and reverse primer, 1 µL ultrapure water and 1 µl cDNA (50 ng). The protocol employed consisted of an enzyme activation step at 95 °C for 2 min, followed by 40 cycles of denaturation at 95 °C for 10 s and annealing at 60 °C for 10 s. A melt gradient step was applied to the end of RT-qPCR reactions, ranging from 65 to 95 °C in 0.5 °C increments.

Relative expression of the genes was calculated using the comparative cycle threshold (Ct) method with *GAPDH* and *18 S* as normalizer genes. The primers employed in this study, as well as the sequences, are listed in Table S1. RT-qPCRs were performed following the “Minimum Information for Publication of Quantitative Real-Time PCR Experiments published by Bustin et al. in 2009^65^.The effect of trypomastigotes of *T. cruzi* over the expression of Rho/Rho kinases in cells was also included as a positive control of the experiment.

### Differential gene expression in Vero cells incubated with EV of *T. cruzi*

In order to confirm and validate transcriptomic results, the differential expression of several genes, including *CD29, PSMC1, PSMD6, MALT1, PLXNC1, UBE2C, SUMO1, SUMO2* and *RABL3* was also analyzed by RT-qPCR, employing *GAPDH* as the normalizer gene. In this case, TaqMan assays were performed with the TaqMan Fast Advanced Master Mix (Thermo Fischer Scientific, USA) following the manufacturer´s protocol. Briefly, each reaction included 5 µL TaqMan Fast Advanced Master Mix (2X), 0.5 µL TaqMan Assay (20X), 3.5 µL ultrapure water and 1 µL cDNA (50 ng). The thermal cycler protocol employed consisted of an initial step at 50 °C for 2 min, followed by an enzyme activation step at 95 °C for 2 min, 40 cycles of denaturation at 95 °C for 3 s and annealing/extending step at 60 °C for 30 s. A melt gradient step was applied to the end of RT-qPCR reactions, ranging from 65 to 95 °C in 0.5 °C increments. TaqMan assay IDs employed for the analysis are listed in Table S2.

### Differential protein expression in Vero cells incubated with EV of *T. cruzi*

The differential expression of distinct proteins in cells incubated with EV of trypomastigotes of *T. cruzi* was also analyzed. In this case, the expression of casein kinase CSNK1G1, a protein of the casein kinase family involved in multiple cellular processes in eukaryotes like cell differentiation, proliferation and apoptosis [69], and srGAP2 (SLIT-ROBO Rho-GTPase-activating protein 2, also known as formin-binding protein 2 FNBP2), a protein that interacts with a novel family of Rho-GTPase activating proteins (GAPs) and inactivates Cdc42 (a member of the Rho-GTPase family involved in regulating the actin cytoskeleton)^21^, was achieved by Western blot. Briefly, Vero cells were grown and incubated with EV of trypomastigotes of the parasite for 4 h. After this time, the cell monolayers were washed using cold sterile PBS and submitted to a lysis step using RIPA buffer (50 mM Tris HCl, 150 mM NaCl, 1.0% NP-40 (v/v), 0.5% sodium deoxycholate (w/v), 1.0 mM EDTA, 0.1% SDS (w/v) and 0.01% sodium azide (w/v), pH 7.4). After the lysis, protein quantification of each sample was performed with the Bradford protein assay (Thermo Fisher Scientific, USA) following the manufacturer´s instructions and the samples were diluted (60 µg per sample) 1:1 in sample buffer^66^, heated for 9 min at 98 °C and subsequently loaded onto 12.5% SDS–polyacrylamide gels for an electrophoretic run for 90 min, using a voltage of 120 V. Once the electrophoresis was completed and an evident separation of the bands in the protein marker was obtained, the proteins in the gel were transferred to PVDF membranes and the Western blot protocol was achieved as described above. In this case, the primary antibodies employed are listed in Table S3, following the dilutions recommended by the manufacturers. Secondary antibodies consisted of goat anti-mouse or goat anti-rabbit IgGs conjugated with peroxidase (1:1000) (Dako Agilent Pathology Solutions, USA). Finally, a loading control was achieved using the anti-GAPDH antibody (1:5000) (Sigma Aldrich, USA).

### Effect of EV of *T. cruzi* over apoptosis

To evaluate the effect of EV of trypomastigotes over apoptosis, 1 × 10^6^ Vero cells were grown onto Nunc 6-well plates (Thermo Fischer Scientific, USA) for 24 h and then preincubated with EV of *T. cruzi* for 2 h. Then, the cell monolayers were washed in MEM and the apoptosis inductor taxol (paclitaxel, an antineoplasic agent) was added at a concentration of 0.1 µg/mL, with the subsequent addition of EV every 8 h during 72 h. After 72 h of culture, the supernatants of each well were removed and cells were harvested using 0.5% EDTA in PBS. The cells were washed using cold sterile PBS and then labeled with fluorescent dyes annexin V and FITC, using an apoptosis detection kit (Trevigen, USA). In this case, 10 µL of binding buffer and 1 µL of annexin V-FITC were added to each sample, mixed gently and incubated for 15 min at room temperature in the dark. Finally, 8 µL of propidium iodide in 400 µL of binding buffer 1X was added just before the analysis, which was performed in a FACS Calibur flow cytometer (BD Biosciences, USA). The results obtained were analyzed using the FlowJo v7.6.5 software (Tree Star Inc., USA).

### Experimental design and data analyses

All experiments were performed in an exploratory manner; thus, *p* values have to be interpreted as descriptive only. The decision to perform 3 independent experiments was made prior to their execution based on the level of variation observed in previous works and sample availability.

Statistical comparisons were performed with repeated-measures one-way or two-way ANOVA or Friedman test using respective post hoc tests for multiple comparisons against specified controls, as recommended by the analysis software and described in the figure legends. Comparisons to a hypothetical value were performed with one sample t test or Wilcoxon signed rank test for means or medians, respectively. All calculations were done using GraphPad Prism 8.4.2 (GraphPad Software, San Diego, CA).

## Supplementary Information

Supplementary Information 1.

Supplementary Information 2.

Supplementary Information 3.

Supplementary Information 4.

Supplementary Information 5.

Supplementary Information 6.

Supplementary Information 7.

## Data Availability

The datasets generated during and analyzed during the current study are available from the corresponding author on reasonable request. In addition, the raw data corresponding to the transcriptome of the vero cells will be available after the publication of the article in the Biosample database belonging to the NCBI, with the BioSample accession SAMN33312896.
