# Author Correction: Extracellular vesicles of trypomastigotes of *Trypanosoma cruzi* induce changes in ubiquitin-related processes, cell-signaling pathways and apoptosis

**DOI:** 10.1038/s41598-023-35751-y

**Published:** 2023-06-06

**Authors:** Alberto Cornet-Gomez, Lissette Retana Moreira, Thales Kronenberger, Antonio Osuna

**Affiliations:** 1 Grupo de Bioquímica y Parasitología Molecular (CTS 183), Departamento de Parasitología, Instituto de Biotecnología, Universidad de Granada, Campus de Fuentenueva, 18071 Granada, Spain; 2 Departamento de Parasitología, Facultad de Microbiología, Universidad de Costa Rica, San José, 11501 Costa Rica; 3 Centro de Investigación en Enfermedades Tropicales (CIET), Universidad de Costa Rica, San José, 11501 Costa Rica; 4 Institute of Pharmacy, Pharmaceutical/Medicinal Chemistry and Tübingen Center for Academic Drug Discovery (TüCAD2), Eberhard Karls University Tübingen, Auf der Morgenstelle 8, 72076 Tübingen, Germany; 5 School of Pharmacy, Faculty of Health Sciences, University of Eastern Finland, 70211 Kuopio, Finland

Correction to: *Scientific Reports* 10.1038/s41598-023-34820-6, published online 10 May 2023

The Acknowledgments section in the original version of this Article was omitted. The Acknowledgments section now reads:

“A.C.G. was supported by the “Beca de Iniciación a la Investigación para estudiantes de Máster” of the University of Granada (Spain). L.R.M. was supported by the Oficina de Asuntos Internacionales y Cooperación Externa (OAICE) of the Universidad de Costa Rica, Ministerio de Ciencia, Tecnología y Telecomunicaciones (MICITT) of the Government of Costa Rica and Fundación Carolina (Spain). TϋCAD2 is funded by the Federal Ministry of Education and Research (BMBF) and the Baden-Württemberg Ministry of Science as part of the Excellence Strategy of the German Federal and State Governments EXC 2180–390900677.

Transmission electron microscopy and atomic force microscopy were performed at the “Centro de Instrumentación Científica”, and nanoparticle tracking analysis was performed at the “Departamento de Física Aplicada” of the Faculty of Sciences, both at the University of Granada. RNA-seq analysis was carried out by AllGenetics & Biology SL (www.allgenetics.eu).

This research was funded by the ERANet program, Research in prevention of congenital Chagas disease: parasitological, placental and immunological markers (ERANet17/HLH-0142 (Cochaco). Instituto Carlos III, Ministerio de Sanidad, Gobierno de España; Fundación Ramón Areces “Interactoma de las exovesículas de T. cruzi y de los inmunocomplejos que forman con las células del hospedador: implicaciones en la patología de la enfermedad de Chagas (2019)”. Ministerio de Ciencia y Tecnología of the government of Spain funded the project PGC2018-099424-B-I00 and the financial support given by the proyect A-BIO-350-UGR18 I+D+i Proyect “Programa Operativo FEDER de Andalucía JJAA” 2014-2020.”

The original Article has been corrected.

