# Supplementary material for: Extracellular vesicles of trypomastigotes of Trypanosoma cruzi induce changes in ubiquitin-related processes, cell-signaling pathways and apoptosis
Source: Sci Rep. 2023 May 10;13:7618. doi: 10.1038/s41598-023-34820-6 (PMC10171165; doi:10.1038/s41598-023-34820-6)
Supplement: Supplementary file 1 — Supplementary Information 1. [file 41598_2023_34820_MOESM1_ESM.docx]

**Figure legend to Supplementary original figures**

Supplementary Figure 1 (Original Figure 1D). Western blot analysis of transialidases in extracellular vesicles of trypomastigotes of *T. cruzi.*

Supplementary Figure 2 (Original Figure 4B) . Western blot analysis of SRGAP3 in a lysate of EVs-stimulated Vero cells (1) and non-stimulated Vero cells (2).

Supplementary Figure 3 (Original Figure 4B and 5B). Western blot analysis of GAPDH in a lysate of EVs-stimulated Vero cells (1) and non-stimulated Vero cells (2).

Supplementary Figure 4 (Original Figure 5B). Western blot analysis of CSNK1G1 in a lysate of EVs-stimulated Vero cells (1) and non-stimulated Vero cells (2).
