# Supplementary figures and images for: Extracellular vesicles of trypomastigotes of Trypanosoma cruzi induce changes in ubiquitin-related processes, cell-signaling pathways and apoptosis
Source: Sci Rep. 2023 May 10;13:7618. doi: 10.1038/s41598-023-34820-6 (PMC10171165; doi:10.1038/s41598-023-34820-6)

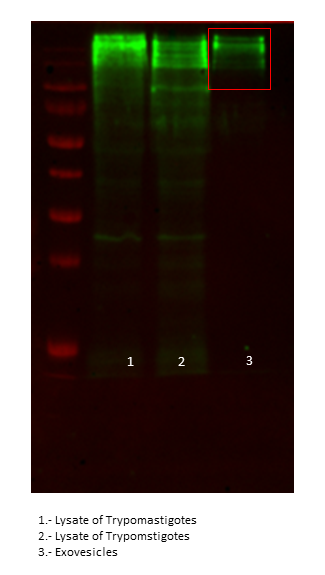

Supplement: Supplementary file 2 — Supplementary Information 2. [file 41598_2023_34820_MOESM2_ESM.tif]

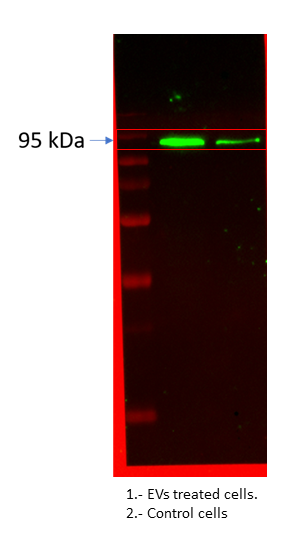

Supplement: Supplementary file 3 — Supplementary Information 3. [file 41598_2023_34820_MOESM3_ESM.tif]

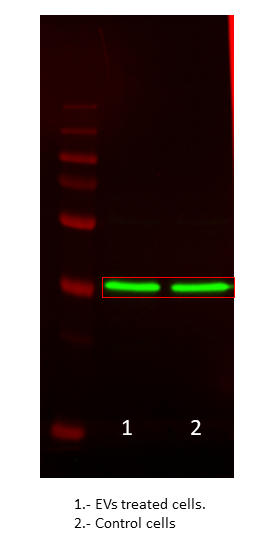

Supplement: Supplementary file 4 — Supplementary Information 4. [file 41598_2023_34820_MOESM4_ESM.tif]

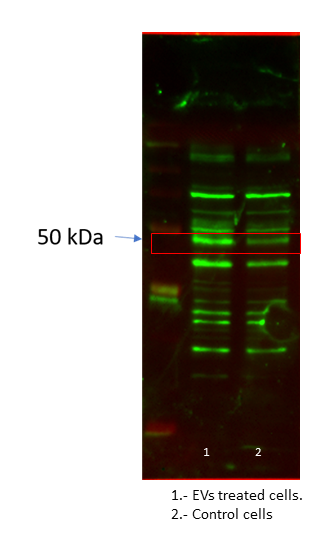

Supplement: Supplementary file 5 — Supplementary Information 5. [file 41598_2023_34820_MOESM5_ESM.tif]

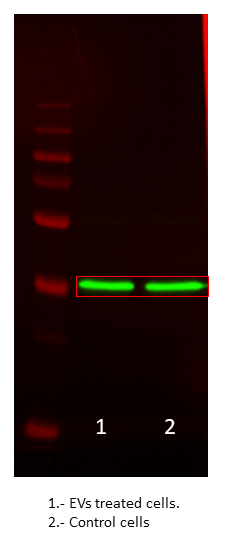

Supplement: Supplementary file 6 — Supplementary Information 6. [file 41598_2023_34820_MOESM6_ESM.tif]
