# Supplementary material for: Extracellular vesicles of trypomastigotes of Trypanosoma cruzi induce changes in ubiquitin-related processes, cell-signaling pathways and apoptosis
Source: Sci Rep. 2023 May 10;13:7618. doi: 10.1038/s41598-023-34820-6 (PMC10171165; doi:10.1038/s41598-023-34820-6)
Supplement: Supplementary file 7 — Supplementary Information 7. [file 41598_2023_34820_MOESM7_ESM.docx]

**Supplementary information**

| **DAVID - GO/KEGG enriched terms with upregulated transcripts** | | | | | | |  |
| --- | --- | --- | --- | --- | --- | --- | --- |
| **Category** | **Term** | **Count** | **PValue** | **FE** | **Bonferroni** | **FDR** | |
| GOTERM_BP_DIRECT | GO:0010467~gene expression | 3 | 0.03 | 11.79 | 1.00 | 1.00 | |
| GOTERM_BP_DIRECT | GO:0023041~neuronal signal transduction | 2 | 0.03 | 62.89 | 1.00 | 1.00 | |
| GOTERM_BP_DIRECT | GO:0035019~somatic stem cell population maintenance | 3 | 0.05 | 8.71 | 1.00 | 1.00 | |
| GOTERM_BP_DIRECT | GO:0030517~negative regulation of axon extension | 2 | 0.06 | 34.30 | 1.00 | 1.00 | |
| GOTERM_BP_DIRECT | GO:0006511~ubiquitin-dependent protein catabolic process | 4 | 0.07 | 4.15 | 1.00 | 1.00 | |
| GOTERM_BP_DIRECT | GO:0042692~muscle cell differentiation | 2 | 0.08 | 25.16 | 1.00 | 1.00 | |
| GOTERM_BP_DIRECT | GO:0043547~positive regulation of GTPase activity | 7 | 0.08 | 2.34 | 1.00 | 1.00 | |
| GOTERM_BP_DIRECT | GO:0030336~negative regulation of cell migration | 3 | 0.09 | 5.96 | 1.00 | 1.00 | |
| GOTERM_BP_DIRECT | GO:0018345~protein palmitoylation | 2 | 0.10 | 18.87 | 1.00 | 1.00 | |
| **Category** | **Term** | **Count** | **PValue** | **FE** | **Bonferroni** | **FDR** | |
| KEGG_PATHWAY | hsa04024:cAMP signaling pathway | 5 | 0.02 | 4.57 | 0.85 | 1.00 | |
| KEGG_PATHWAY | hsa05031:Amphetamine addiction | 3 | 0.05 | 8.23 | 0.99 | 1.00 | |
| KEGG_PATHWAY | hsa04925:Aldosterone synthesis and secretion | 3 | 0.07 | 6.70 | 1.00 | 1.00 | |
| KEGG_PATHWAY | hsa04911:Insulin secretion | 3 | 0.08 | 6.39 | 1.00 | 1.00 | |
| KEGG_PATHWAY | hsa04912:GnRH signaling pathway | 3 | 0.09 | 5.97 | 1.00 | 1.00 | |
|  |  |  |  |  |  |  | |
| **DAVID - GO/KEGG enriched terms with downregulated transcripts** | | | | | | |  |
| **Category** | **Term** | **Count** | **PValue** | **FE** | **Bonferroni** | **FDR** | |
| GOTERM_BP_DIRECT | GO:0050852~T cell receptor signaling pathway | 6 | 0.00 | 7.40 | 0.62 | 0.86 | |
| GOTERM_BP_DIRECT | GO:0016569~covalent chromatin modification | 5 | 0.00 | 8.08 | 0.92 | 0.86 | |
| GOTERM_BP_DIRECT | GO:0070373~negative regulation of ERK1 and ERK2 cascade | 4 | 0.00 | 12.59 | 0.95 | 0.86 | |
| GOTERM_BP_DIRECT | GO:0048208~COPII vesicle coating | 4 | 0.00 | 11.97 | 0.97 | 0.86 | |
| GOTERM_BP_DIRECT | GO:0006890~retrograde vesicle-mediated transport, Golgi to ER | 4 | 0.01 | 8.90 | 1.00 | 1.00 | |
| GOTERM_BP_DIRECT | GO:0000165~MAPK cascade | 6 | 0.01 | 4.18 | 1.00 | 1.00 | |
| GOTERM_BP_DIRECT | GO:0038095~Fc-epsilon receptor signaling pathway | 5 | 0.02 | 5.13 | 1.00 | 1.00 | |
| GOTERM_BP_DIRECT | GO:0042059~negative regulation of epidermal growth factor receptor signaling pathway | 3 | 0.02 | 15.21 | 1.00 | 1.00 | |
| GOTERM_BP_DIRECT | GO:0045944~positive regulation of transcription from RNA polymerase II promoter | 12 | 0.02 | 2.23 | 1.00 | 1.00 | |
| GOTERM_BP_DIRECT | GO:0002223~stimulatory C-type lectin receptor signaling pathway | 4 | 0.02 | 6.95 | 1.00 | 1.00 | |
| GOTERM_BP_DIRECT | GO:0072108~positive regulation of mesenchymal to epithelial transition involved in metanephros morphogenesis | 2 | 0.02 | 91.26 | 1.00 | 1.00 | |
| GOTERM_BP_DIRECT | GO:0090263~positive regulation of canonical Wnt signaling pathway | 4 | 0.03 | 6.08 | 1.00 | 1.00 | |
| GOTERM_BP_DIRECT | GO:0048812~neuron projection morphogenesis | 3 | 0.03 | 11.41 | 1.00 | 1.00 | |
| GOTERM_BP_DIRECT | GO:0048194~Golgi vesicle budding | 2 | 0.03 | 60.84 | 1.00 | 1.00 | |
| GOTERM_BP_DIRECT | GO:0043066~negative regulation of apoptotic process | 7 | 0.04 | 2.81 | 1.00 | 1.00 | |
| GOTERM_BP_DIRECT | GO:0032091~negative regulation of protein binding | 3 | 0.04 | 9.61 | 1.00 | 1.00 | |
| GOTERM_BP_DIRECT | GO:0035148~tube formation | 2 | 0.04 | 45.63 | 1.00 | 1.00 | |
| GOTERM_BP_DIRECT | GO:0043087~regulation of GTPase activity | 3 | 0.05 | 8.42 | 1.00 | 1.00 | |
| GOTERM_BP_DIRECT | GO:0038061~NIK/NF-kappaB signaling | 3 | 0.05 | 8.30 | 1.00 | 1.00 | |
| GOTERM_BP_DIRECT | GO:0051436~negative regulation of ubiquitin-protein ligase activity involved in mitotic cell cycle | 3 | 0.05 | 7.71 | 1.00 | 1.00 | |
| GOTERM_BP_DIRECT | GO:0043123~positive regulation of I-kappaB kinase/NF-kappaB signaling | 4 | 0.06 | 4.53 | 1.00 | 1.00 | |
| GOTERM_BP_DIRECT | GO:0090090~negative regulation of canonical Wnt signaling pathway | 4 | 0.06 | 4.48 | 1.00 | 1.00 | |
| GOTERM_BP_DIRECT | GO:0007030~Golgi organization | 3 | 0.06 | 7.40 | 1.00 | 1.00 | |
| GOTERM_BP_DIRECT | GO:0034498~early endosome to Golgi transport | 2 | 0.06 | 30.42 | 1.00 | 1.00 | |
| GOTERM_BP_DIRECT | GO:0019068~virion assembly | 2 | 0.06 | 30.42 | 1.00 | 1.00 | |
| GOTERM_BP_DIRECT | GO:0051437~positive regulation of ubiquitin-protein ligase activity involved in regulation of mitotic cell cycle transition | 3 | 0.06 | 7.20 | 1.00 | 1.00 | |
| GOTERM_BP_DIRECT | GO:0016477~cell migration | 4 | 0.07 | 4.24 | 1.00 | 1.00 | |
| GOTERM_BP_DIRECT | GO:0031145~anaphase-promoting complex-dependent catabolic process | 3 | 0.07 | 6.93 | 1.00 | 1.00 | |
| GOTERM_BP_DIRECT | GO:0048193~Golgi vesicle transport | 2 | 0.07 | 26.07 | 1.00 | 1.00 | |
| GOTERM_BP_DIRECT | GO:1901800~positive regulation of proteasomal protein catabolic process | 2 | 0.08 | 22.82 | 1.00 | 1.00 | |
| GOTERM_BP_DIRECT | GO:0035855~megakaryocyte development | 2 | 0.08 | 22.82 | 1.00 | 1.00 | |
| GOTERM_BP_DIRECT | GO:0043547~positive regulation of GTPase activity | 7 | 0.09 | 2.26 | 1.00 | 1.00 | |
| GOTERM_BP_DIRECT | GO:0060071~Wnt signaling pathway, planar cell polarity pathway | 3 | 0.09 | 5.95 | 1.00 | 1.00 | |
| GOTERM_BP_DIRECT | GO:0010596~negative regulation of endothelial cell migration | 2 | 0.09 | 20.28 | 1.00 | 1.00 | |
| GOTERM_BP_DIRECT | GO:0050870~positive regulation of T cell activation | 2 | 0.09 | 20.28 | 1.00 | 1.00 | |
| GOTERM_BP_DIRECT | GO:0043161~proteasome-mediated ubiquitin-dependent protein catabolic process | 4 | 0.10 | 3.60 | 1.00 | 1.00 | |
| **Category** | **Term** | **Count** | **PValue** | **FE** | **Bonferroni** | **FDR** | |
| KEGG_PATHWAY | hsa04810:Regulation of actin cytoskeleton | 4 | 0.10 | 3.54 | 1.00 | 1.00 | |

**Supplementary Table S1.** Top ranked KEGG/GO processes detected by enrichment analyses on all up- and downregulated DEGs (ranked by *p*-values).


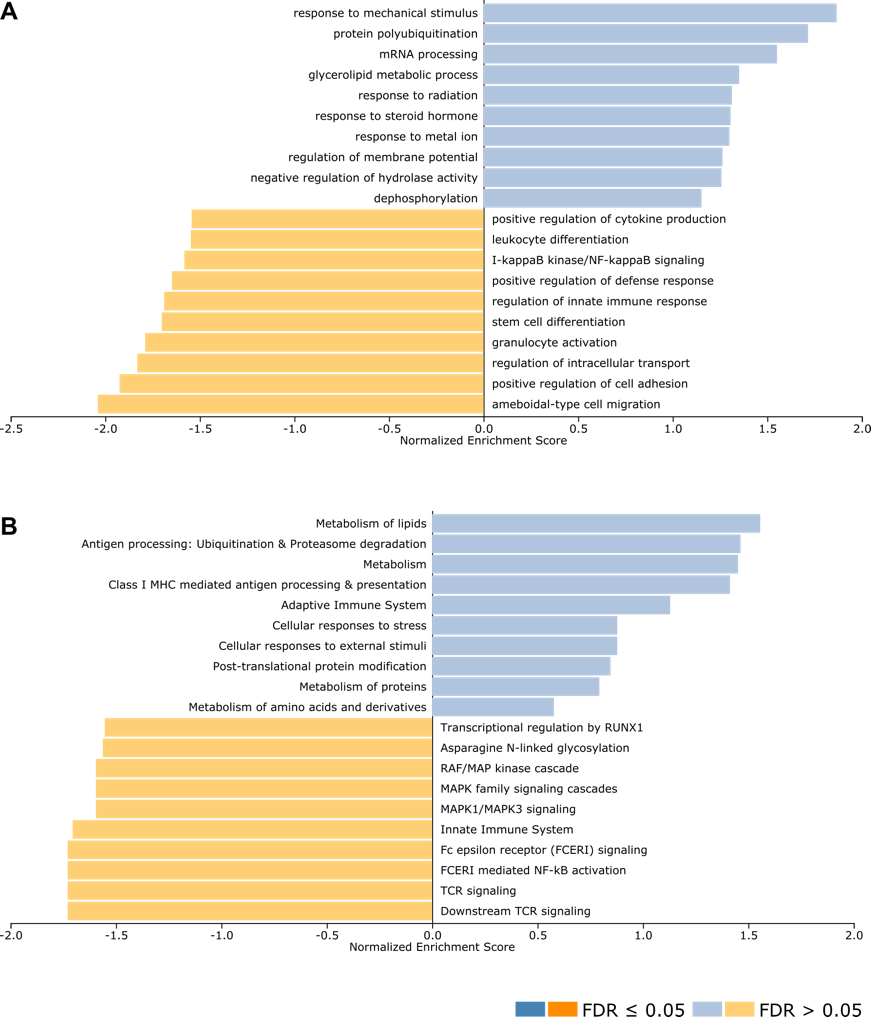


**Supplementary Figure S1.** Gene set enrichment analyses for gene ontology terms (A) and pathways (B)

| **Gene** | **Sequence** | **Expected size (bp)** |
| --- | --- | --- |
| *RhoA* | F: 5´-TTCCATCGACAGCCCTGATAGTTTA-3´  R: 5´-CACGTTGGGACAGAAATGCTTG-3´ | 179 |
| *Rac1* | F: 5´-TGCAAAGTGGTATCCTGAGGTGCG-3´  R: 3´- GCGGATAGGTGATGGGAGTCATCAGC-5´ | 122 |
| *Cdc42* | F: 5´-CCTCCAGAACCGAAGAAGAGCCG-3´  R: 3´-AGTGCATGTGGGTAGGTGCAGGG-3´ | 178 |
| *GAPDH* | F: 5´-GCAAGTTCCATGGCACCGTGAAG-3´  R: 3´-AGGCGTTGCTGACGATCTTGAGGC-3´ | 239 |
| *18S* | F: 5´-GTAACCCGTTGAACCCCATT-3´  R: 3´-CCATCCAATCGGTAGTAGCG-3´ | 151 |

**Supplementary Table S2.** Primers and sequences employed for the expression analysis of Rho GTPases in this study

| **Gene** | **Assay ID** |
| --- | --- |
| *CD29*  *PSMC1*  *PSMD6*  *MALT1* | Hs01127536_m1  Hs02386942_g1  Hs01030737_m1  Hs01120060_m1 |
| *PLXNC1*  *UBE2C*  *SUMO1*  *SUMO2*  *RABL3*  *NRXN1*  *GAPDH* | Hs00194968_m1  Hs00964100_g1  Hs02339312_g1  Hs02743873_g1  Hs00378653_m1  Hs00611317_m1  Hs02786624_g1 |

**Supplementary Table S3.** TaqMan Assay IDs employed for the validation analysis of different genes in this study

| **Antibody** | **Dilution** |
| --- | --- |
| Rabbit anti-human mouse CSNK1G1 Polyclonal antibody  (Mybiosource, USA)  Rabbit anti-human SRGAP3 polyclonal antibody  (Mybiosource, USA)  Anti-GAPDH (Loading control antibody, GA1R)  (Thermo Fischer Scientific, USA) | 1:1,000  1:1,000  1:5,000 |

**Supplementary Table S4.** Primary antibodies and dilutions employed for differential protein expression analysis of cells incubated with EVs of trypomastigotes of *T. cruzi*
